# Pterostilbene Protects against Osteoarthritis through NLRP3 Inflammasome Inactivation and Improves Gut Microbiota as Evidenced by *In Vivo* and *In Vitro* Studies

**DOI:** 10.1021/acs.jafc.3c09749

**Published:** 2024-04-16

**Authors:** Yen-Chien Lee, Yu-Ting Chang, Yung-Hsuan Cheng, Rosita Pranata, Heng-Hsuan Hsu, Yen-Lin Chen, Rong-Jane Chen

**Affiliations:** † Department of Oncology, Tainan Hospital, Tainan 70043, Taiwan; ‡ Department of Internal Medicine, National Cheng Kung University Hospital, College of Medicine, Tainan 70043, Taiwan; § Department of Nursing, National Tainan Junior College of Nursing, Tainan 70043, Taiwan; ∥ Department of Food Safety/Hygiene and Risk Management, College of Medicine, National Cheng Kung University, Tainan 701, Taiwan; ⊥ Department of Environmental and Occupational Health, College of Medicine, National Cheng Kung University, Tainan 701, Taiwan; # Bioresource Collection and Research Center (BCRC), Food Industry Research and Development Institute, Hsinchu 300, Taiwan

**Keywords:** osteoarthritis, pterostilbene, NLRP3 inflammasome, cartilage catabolism, gut microbiota

## Abstract

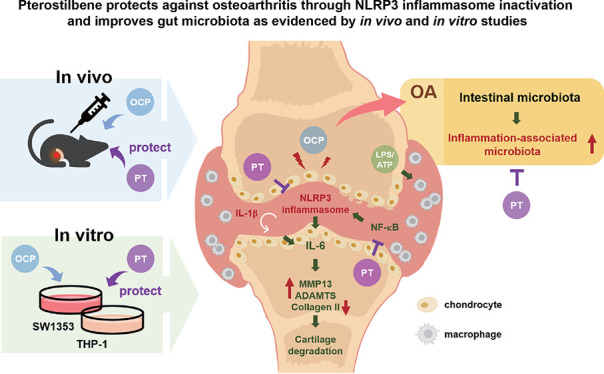

Osteoarthritis (OA) is a persistent inflammatory disease, and long-term clinical treatment often leads to side effects. In this study, we evaluated pterostilbene (PT), a natural anti-inflammatory substance, for its protective effects and safety during prolonged use on OA. Results showed that PT alleviated the loss of chondrocytes and widened the narrow joint space in an octacalcium phosphate (OCP)-induced OA mouse model (n = 3). *In vitro* experiments demonstrate that PT reduced NLRP3 inflammation activation (relative protein expression: C: 1 ± 0.09, lipopolysaccharide (LPS): 1.14 ± 0.07, PT: 0.91 ± 0.07, LPS + PT: 0.68 ± 0.04) and the release of inflammatory cytokines through NF-κB signaling inactivation (relative protein expression: C: 1 ± 0.03, LPS: 3.49 ± 0.02, PT: 0.66 ± 0.08, LPS + PT: 2.78 ± 0.05), ultimately preventing cartilage catabolism. Interestingly, PT also altered gut microbiota by reducing inflammation-associated flora and increasing the abundance of healthy bacteria in OA groups. Collectively, these results suggest that the PT can be considered as a protective strategy for OA.

## Introduction

Osteoarthritis (OA) is a common degenerative joint disease characterized by mild inflammation and high clinical heterogeneity.^1^ The clinical phenotypes of OA, including chronic pain, inflammation, metabolic syndrome, bone and cartilage catabolism, mechanical overload, and minimal joint disease, contribute to the challenges suffered by patients.^1^ Those with OA often endure persistent pain, leading to poor quality of life and disability.^2^ According to the latest report from the World Health Organization (WHO), OA had affected more than 528 million people worldwide in 2019, especially in the middle-aged and elderly.^3^ Moreover, with the increasing aging population, OA has garnered more attention than ever before.

The constitutive mechanism of OA is complex and involves multiple factors, such as the accumulation of advanced glycation end products (AGEs), deposition of uric acid crystals, high-intensity physical activity, and joint damage.^1^ The joint, which connects two adjacent bones, is covered with a special articular cartilage layer composed of extracellular matrix components containing type II collagen, aggrecan, or other proteoglycans.^4^ The extracellular matrix provides the tissue with integrity and elasticity, playing a role in regulating cell proliferation and differentiation.^5^ Chondrocytes, the major cell type of articular cartilage, play a crucial role in maintaining the balance between the extracellular matrix and tissue homeostasis.^6^ Upon stimulation, chondrocytes change phenotype and express a variety of inflammatory factors, such as cytokines, chemokines, alarmins, damage-associated molecular patterns (DAMPs), and adipokines, resulting in the gradual disappearance of cartilage.^4^ Metalloproteases (MMPs) and A Disintegrin and Metalloproteinase with Thrombospondin motifs (ADAMTS) families are the major cartilage matrix-degrading enzymes capable of degrading proteoglycan.^7,8^ The disrupted balance between cartilage-decomposing and cartilage-synthesizing proteins leads to OA.^4^

Catabolic cytokines and chemokines produced by chondrocytes are associated with NF-κB activation, resulting in reduced collagen synthesis and increased release of inflammatory cytokines, including TNF-α, IL-1β, and IL-6.^9^ The NF-κB signaling pathway is known to mediate upstream of NLRP3 inflammasomes’ function, which also induces degrading enzymes like MMP-3 and MMP-13, leading to cartilage degeneration and synovial inflammation.^10,11^ NLRP3 inflammasomes comprise the receptor protein NLRP3, ASC, and pro-caspase-1, inducing the activation of caspase-1 to degrade pro-IL-1β or pro-IL-18 into a mature form, leading to an inflammatory response.^12^ These inflammatory cytokines recruit more inflammatory cells, such as macrophages, that infiltrate into the joints to communicate with chondrocytes. The accumulated inflammatory responses initiate more proinflammatory cytokine release, forming a vicious circle and affecting the entire OA process.^13^ In this context, identifying the molecular profiles would help to reveal the major inflammatory mechanisms of OA and the development of molecular-based treatment strategies.

Because of the complex mechanism of action, the current drug treatment of OA is mainly focused on relieving symptoms, and there are only a low number of treatments without side effects available.^14^ For instance, common medications like NSAIDs might induce organ damage with long-term consumption;^15^ corticosteroids provide only temporary pain relief and may cause side effects on cartilage;^16^ overuse of analgesics has also been associated with cartilage toxicity.^17^ Considering safety and advantageous treatments for OA, the nontoxic nature of natural substances is one of the first choices. Recent studies have found that food rich in polyphenols could protect against age-related diseases, such as arthritis and osteoporosis.^18^ Pterostilbene (PT) has been well-studied as a promising dietary polyphenolic compound with various chemopreventive and chemotherapeutic effects, including anti-inflammation, anticancer, and antifibrosis.^19−23^ Various studies of specific organs and diseases have reported the benefits of PT. For example, PT treatment has been shown to inhibit NLRP3 inflammasome activation and fibrosis in kidney tubular cells, thus attenuating chronic kidney disease (CKD).^12^ Additionally, PT has been found to attenuate the neuroinflammatory response characteristic of Alzheimer’s disease (AD) by inhibiting the NLRP3/caspase-1 inflammasome pathway.^24^ Furthermore, PT has been reported to improve nonalcoholic fatty liver disease (NAFLD) by reducing lipid accumulation and inflammation.^25^ Recently, the effect of PT on bone homeostasis has been reported, demonstrating that pterostilbene-isothiocyanate suppresses osteoclastogenesis and promotes osteogenesis to ameliorate osteoporosis.^26^ In addition, PT has been found to inhibit ROS generation and inflammation, exerting an antiarthritic effect on rheumatoid arthritis (RA).^27^ Based on these findings, PT could be a potential strategy for the prevention and therapy of OA.

To investigate the possible preventive and therapeutic effects of PT on OA, we performed a variety of inflammatory environments to mimic the influence of OA progression. Furthermore, in addition to local inflammation, systemic inflammation has also been reported to play a role in OA.^28^ For instance, obesity not only is an important risk factor for OA progression due to the increased mechanical load on the knee joint but also perturbs the intestinal microbiota, leading to a persistent and low-grade inflammatory response.^28^ Therefore, this study not only demonstrated a novel role and detailed underlying molecular mechanism of PT on NLRP3 inflammasome-dependent inflammation in OA but also displayed improvement of gut microbiota in OA through PT treatment.

## Materials and Methods

### Chemicals and Reagents

Octacalcium phosphate crystals (OCP), a type of basic calcium phosphate (BCP) often deposited in joints, causing acute inflammatory arthritis and joint degeneration,^29^ were used to induce OA in this study. OCP precipitate was collected from dicalcium phosphate (Sigma-Aldrich, Darmstadt, Germany) and 0.05 M diammonium phosphate solution (Sigma-Aldrich, Darmstadt, Germany) according to a previous study.^30^ The OCP powder collected after precipitation was washed with distilled water and dried at 37 °C. Pterostilbene (PT) with a purity of 98% was purchased from Combi Blocks (San Diego, USA). LPS and ATP were purchased from Sigma-Aldrich (Darmstadt, Germany).

### *In Vivo* Experiments

Twelve week old female C57BL/6 mice were purchased from the National Laboratory Animal Center (Taipei, Taiwan). All animal experiments were approved and followed the guidelines of the Institutional Animal Care and Use Committee (IACUC) of the Laboratory Animal Center in National Cheng Kung University Medical College (Approval No. 107204). Mice were housed at 24 ± 2 °C and 50 ± 10% humidity with a 12 h light/dark cycle and randomly divided into groups. Mice were acclimatized for 7 days before the start of the experiment.

The mice were randomly divided into four groups: control, the OCP group, OCP + PT 100 (100 mg/kg PT), and OCP + PT 200 (200 mg/kg PT), with three mice in each group. OA was induced by the use of an OCP (20 μg), which was suspended in PBS and injected into the right knee of the mice on days 1 and day 14. PT was dissolved in corn oil and administered by oral gavage for 28 days at 100 and 200 mg/kg. After the mice were sacrificed, the kidneys, livers, and knees were collected for staining analysis. In the specimen processing section, the knees were soaked in an EDTA solution for decalcification after formaldehyde treatment. The concentrations of biochemical indicators, such as GOT, GPT, blood urea nitrogen (BUN), and creatinine (CRE), were detected by an automated clinical chemistry analyzer (FUJI DRI-CHEM 4000i, Tokyo, Japan).

### Hematoxylin and Eosin Staining

Hematoxylin and eosin staining (H&E) (Merck, Catalog Nos. 105175 and 102439, Darmstadt, Germany) was used to evaluate the histopathological structure of the kidney and liver tissues. Samples were dehydrated through an alcohol gradient and stained with hematoxylin for 2 min. After the excess dye was washed away, samples were stained with eosin for 2 min. Slides were dehydrated and sealed for observation by optical electron microscopy.

### Safranin O and Fast Green Staining

Safranin O and Fast Green staining (ScienCell, Catalog No. 8348, Carlsbad, USA) was used to distinguish cartilage tissue from bone tissue. Mice knee joints were fixed in 4% paraformaldehyde for 24 h at 4 °C and decalcified with 10% EDTA (Sigma-Aldrich, Darmstadt, Germany) for 2 weeks at room temperature. Samples were dehydrated through an alcohol gradient, stained with 0.1% Fast Green for 10 min, and soaked in 1% acetic acid for a few seconds. Samples were then stained with 0.5% Safranin O for 50 min. Finally, the slides were dehydrated and sealed, and the morphology was observed under an optical electron microscope (Nikon ECLIPSE E600, Nikon, Tokyo, Japan).

### Immunohistochemistry Staining

The decalcified bone tissue was dehydrated through an alcohol gradient. After soaking in citric acid (pH 6.0) to present the antigen, the samples were treated with 3% H_2_O_2_ gently for 10 min and then washed with PBS. Following the manufacturer’s guide (IHC Select, Merck, Darmstadt, Germany), samples were sequentially blocked, hybridized with primary and secondary antibodies, and covered with streptavidin HRP. Finally, samples were appropriately stained with DAB chromogen and hematoxylin. The slides were dehydrated and mounted for observation by optical electron microscopy.

### Gut Microbiota Analysis

The analysis of gut microbiota in animal studies was conducted at Tri-I Biotech Inc. (Taipei, Taiwan). DNA was extracted from the feces samples using the QIAamp Fast DNA Stool Mini Kit (Qiagen, Catalog No. 51604, Hilden, Germany). The concentration was determined using Qubit, and primers 341F and 805R were used to amplify the 16S rDNA V3–V4 region. The amplified PCR product was purified with the QIAquick PCR Purification Kit (Qiagen, Catalog No. 28106, Hilden, Germany). After pooling, the mixed samples were further purified with AMPure XP beads (Beckman Coulter, CA, USA) and were subjected to electrophoresis with 2% agarose. After another round of purification with the MinElute Gel Extraction Kit (Qiagen, Catalog No. 28604, Hilden, Germany), the Celero DNA-Seq System (1-96) (NuGEN, CA, USA) was used to construct DNA into a library, which was then sequenced using an Illumina MiSeq System (Illumina, CA, USA). Based on the sequence data, the strains were classified by the obtained OTU (operational taxonomic unit).

### Cell Culture

Human SW1353 chondrocytes and human THP-1 monocytes were purchased from the Bioresource Collection and Research Center (Food Industry Research and Development Institute, Hsinchu, Taiwan). Human SW1353 chondrocytes were cultured in Dulbecco’s modified Eagle’s medium (DMEM) (Gibco, Thermo Fisher Scientific, MA, USA), and human THP-1 monocytes were cultured in RPMI 1640 medium (Simply Biologics, Miaoli, Taiwan). Both media were supplemented with 10% (v/v) fetal bovine serum (FBS) (Gibco, Thermo Fisher Scientific Inc., MA, USA), 1% antibiotic–antimycotic solution, or 1% sodium pyruvate solution. Cells were cultured at 37 °C in an atmosphere of 95% humidified air and 5% CO_2_.

### Cell Viability Assay

Cell viability was assessed using the 3-[4,5-dimethylthiazole-2-yl]-2,5-diphenyltetrazolium bromide (MTT) assay. SW1353 cells were seeded in 96-well plates and pretreated with different concentrations of PT (5, 7.5, or 10 μM) for 2 h followed by treatment with IL-1β (10 ng/mL) for 24 h. After exposure, the cells were washed with phosphate-buffered saline (PBS) and incubated in MTT solution (5 mg/mL) for 2 h at 37 °C. The supernatants were discarded, and the crystals were dissolved by DMSO. Absorbance was measured at 570 nm using a spectrophotometer.

### Cytokine Detection

The concentration of cytokines (IL-6 and IL-1β) in the cell supernatants was determined using commercial Human Quantikine ELISA Kits (R&D, Thermo Fisher Scientific, Catalog No. DTA00D, MA, USA). SW1353 cells were treated as described above. THP-1 cells were pretreated with PT (10 μM) for 2 h and stimulated by lipopolysaccharide (LPS) and adenosine triphosphate (ATP) (LPS 1 μg/mL and ATP 5 mM) for 24 h. Supernatants were collected and diluted following the guidelines provided by the manufacturer. Optical density values were measured at a wavelength of 595 nm.

### Western Blot Analysis

Cells were harvested and lysed to isolate the proteins. Quantified proteins were loaded onto sodium dodecyl sulfate-polyacrylamide gel electrophoresis (SDS-PAGE) and transferred to polyvinylidene difluoride (PVDF) membranes. After blocking with 5% nonfat milk, the membranes were incubated with various primary antibodies. ADAMTS-5 (#Ab41037), collagen II (#Ab185430), MMP-13 (#Ab39012), and NLRP3 (#Ab263899) antibodies were purchased from Abcam (Cambridge, UK); caspase-1 (#24232), IL-1β (#12242S), phospho-NF-κB (#3033), NF-κB (#8242), and GAPDH (#5174) antibodies were purchased from Cell Signaling Technology (MA, USA); and GADPH antibody was purchased from Epitomics (CA, USA). Subsequently, the membranes were washed in Tris-buffered saline with 0.1% Tween 20 detergent (TBST) and incubated with secondary antibodies. Protein expression was detected using enhanced chemiluminescent reagents (Invitrogen; Thermo Fisher Scientific, MA, USA) and analyzed using iBright Imaging Systems (iBright FL 1000; Thermo Fisher Scientific, MA, USA).

### RNA Extraction and Real-Time Quantitative Polymerase Chain Reaction (RT-qPCR)

THP-1 cells were pretreated with PT (10 μM) for 2 h and stimulated by LPS (1 μg/mL) for 24 h. THP-1 cells were subsequently mixed with NecleoZOL to isolate RNA. Total RNA was quantified using a NanoDropTW 1000 Spectrophotometer (Thermo Fisher Scientific, MA, USA). A specific amount of RNA was used to synthesize cDNA with the MMLV reverse transcription kit (Thermo Fisher Scientific, Catalog No. 28025013, MA, USA). RT-qPCR was conducted using a FastStart Universal SYBR Green Master in the ABI Step One Plus Real-Time PCR System (Thermo Fisher Scientific, MA, USA). Primers were purchased from Genomics (Taipei, Taiwan), and their details are described in Table 1.

**Table 1:** RT-qPCR Primer Sequences Used in the Study

primer	sequence (5′ to 3′)	
IL-1β (human)	F	CAGCTACGAATCTCCGACCAC
	R	GGCAGGGAACCAGCATCTTC
TNF-α (human)	F	ATGTGCTCCTCACCCACACC
	R	GTCGGTCACCCTTCTCCAGCT
IL-6 (human)	F	AGCCACTCACCTCTTCAGAAC
	R	GCCTCTTTGCTGCTTTCACAC

### Statistical Analysis

The statistical analysis was presented using the commercial statistical software SigmaPlot 10.0. Differences between groups were analyzed statistically using the two-tailed Student’s *t* test. All data were expressed as mean ± standard deviation, and significant differences were expressed as *^/#^*p* < 0.05.

## Results

### PT Ameliorates the Cartilage Matrix Loss and the Joint Damage in the OCP-Induced OA Model

**Figure 1:**
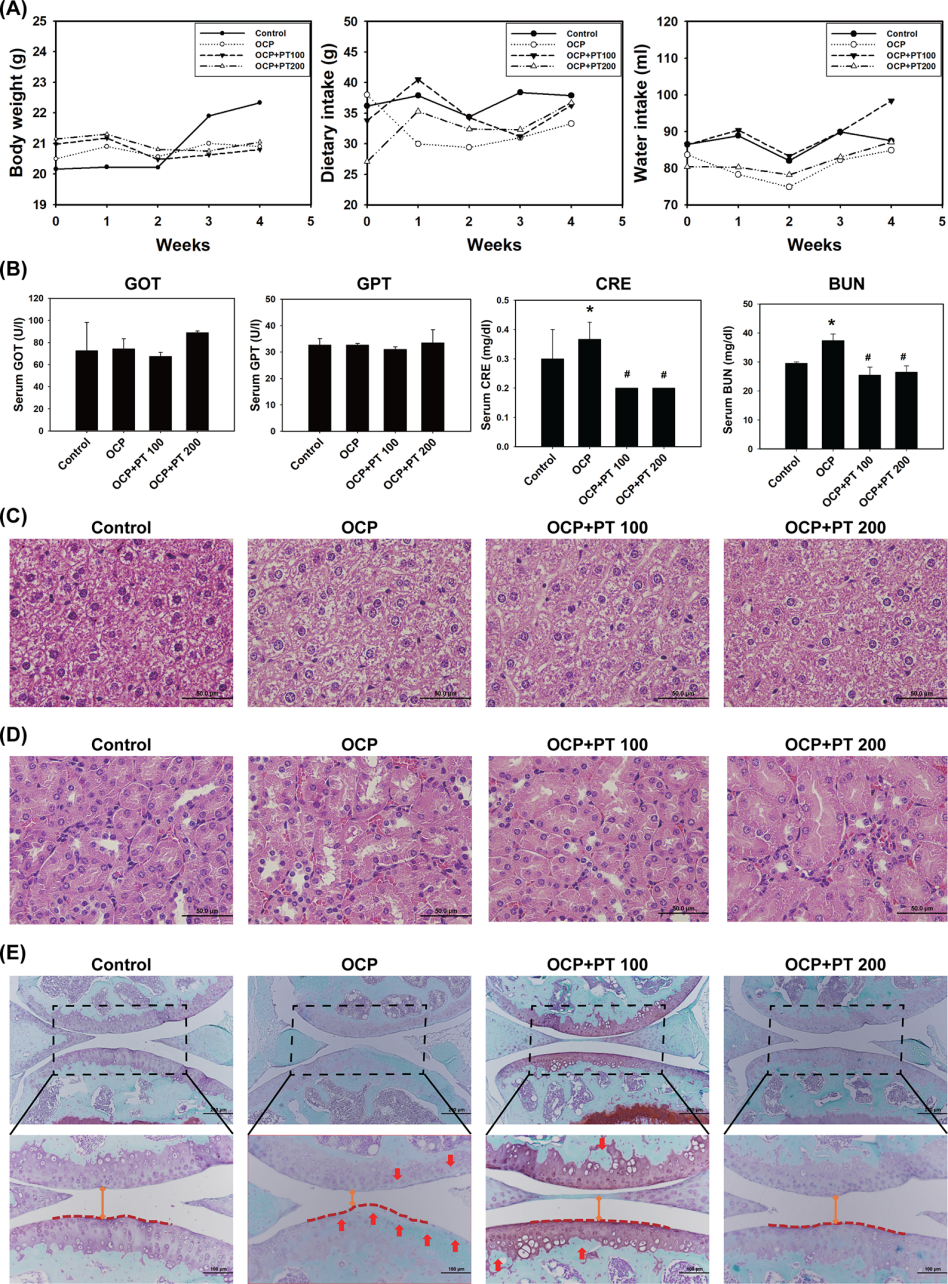
PT improved joint space narrowing and cartilage loss in the OCP-induced OA model. Female C57BL/6 mice were divided into four groups, each consisting of three mice: control, OCP, OCP + PT 100 (mg/kg), and PT 200 (mg/kg). Mice in the control group were injected with PBS in both knees, whereas those in the OCP, OCP + PT 100, and OCP + PT 200 groups were injected with PBS in the left knee and OCP in the right knee. The mice in the OCP + PT 100 and the OCP + PT 200 groups were then fed daily with 100 and 200 mg PT/kg body weight, respectively, for 4 weeks. (A) Body weight, dietary intake, and water intake of mice in control, OCP, OCP + PT 100, and OCP + PT 200 groups. (B) Serum biochemical parameters of GOT, GPT, CRE, and BUN were measured. All data are presented as the mean ± SD from three independent experiments. *p < 0.05 compared with the control group; ^#^compared with the OCP group. (C) H&E staining results of liver and (D) kidney tissues (scale bar: 50 μm). (E) Safranin O and Fast Green staining of joint tissues showed joint space narrowing (orange line segment), marginal bone unevenness (dark red dotted line), and articular cartilage erosion (red arrow) (original magnification: ×10 and ×20, scale bar: 200, 100 μm).

Our previous studies reported the anti-inflammatory effects of PT through *in vitro* and *in vivo* experiments.^12,31^ Clinically relevant models are essential for a deeper understanding of the disease’s impact and for assessing the efficacy of treatment compounds.^32^ Accordingly, we began by investigating the protective effect of PT at a physiological level using an OA mouse model in this study. OCP-induced OA mice were treated with PT 100 (100 mg/kg) and PT 200 (200 mg/kg) separately. We first determined whether PT caused a burden on mice and other organs. The results showed no significant changes in body weight, daily diet, and water intake in mice during OCP and PT-combined treatment (Figure 1A). Serum biochemical parameters and histopathological assessment of the liver and kidney showed no differences between the control and PT-combined groups (Figure 1B–D). However, serum CRE and BUN levels in the OCP groups were increased compared to the control groups, whereas PT-combined treatment reduced serum CRE and BUN levels compared to those in the OCP treatment groups (Figure 1B). In addition, the morphological observation of cartilage tissue and bone exhibited cartilage matrix degradation in the OCP group (Figure 1E). In the OCP group, the surface of the subchondral bone was uneven with a narrow bone space, and the cartilage matrix was lost and corroded. However, the surface of the subchondral bone became smooth with a wide bone space, and the cartilage matrix was recovered after PT-combined treatment (Figure 1E). These results confirm the protective effect of PT on OCP-induced OA, preventing damage and attenuating OA development.

### PT Inhibits IL-1β-Induced IL-6 Production and Prevents Cartilage Extracellular Matrix Degradation in SW1353 Cells

**Figure 2:**
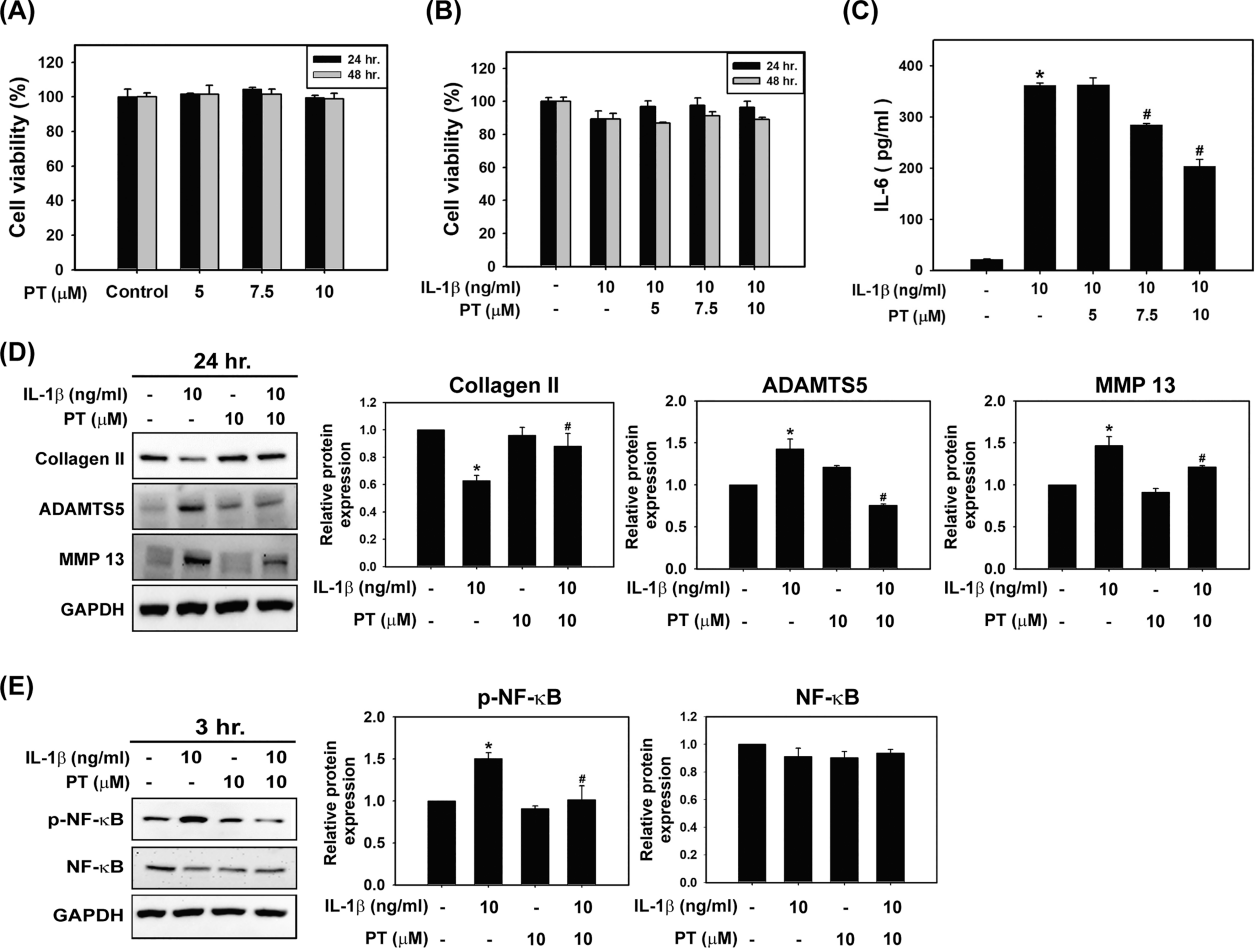
PT reduced the level of IL-6 and prevented IL-1β-induced cartilage degradation in SW1353 cells. (A) Cell viability was measured by MTT assay in SW1353 cells treated with PT (5, 7.5, and 10 μM) or (B) PT (10 μM) and IL-1β (10 ng/mL) for 24 and 48 h. (C) The IL-6 concentration was detected by ELISA after treatment with PT (5, 7.5, and 10 μM) or IL-1β (10 ng/mL). (D) SW1353 cells were treated with PT (10 μM) and/or with IL-1β (10 ng/mL) for 24 h. The protein expression of collagen II, ADAMTS5, and MMP-13 was detected by Western blot analysis, and their respective quantifications are presented. GAPDH was used as a loading control (*n* = 3). (E) SW1353 cells were treated with PT (10 μM) and/or with IL-1β (10 ng/mL) for 3 h. The protein expression of the phosphorylated NF-κB and NF-κB was detected by Western blot analysis, and their respective quantifications are shown. GAPDH was used as a loading control (*n* = 3). All data are presented as mean ± SD from three independent experiments. *p < 0.05 compared with the control groups. ^#^*p* < 0.05 compared with IL-1β treated groups.

Previous studies have indicated the involvement of various inflammatory cytokines in OA inflammation.^13,33,34^ IL-6 is one of the inflammatory cytokines whose level is elevated in the serum and synovial fluid of OA patients, contributing to MMP production and cartilage destruction.^34^ To explore the protective mechanism of PT in OA, we examined the production of IL-6 in human SW1353 chondrocytes. IL-1β, a crucial proinflammatory cytokine produced during the development of OA, was used to mimic the inflammatory condition, combined with PT treatment in the study by using SW1353 cells. In Figure 2A, cell viability showed no significant difference in PT-treated groups compared to control groups in SW1353 cells after treatment for 24 and 48 h, indicating that the concentration of PT used in SW1353 cells did not induce cytotoxicity. Figure 2B shows that IL-1β (10 ng/mL) treatment alone slightly decreased cell viability, which was restored by PT treatment. Furthermore, the result further demonstrated that IL-6 release increased with IL-1β treatment, whereas pretreatment with PT decreased the production of IL-6 in a dose-dependent manner (Figure 2C). In the cartilage catabolism pathways, the cartilage-decomposing proteins MMP-13 and ADAMTS-5 were upregulated, whereas the cartilage-synthetic protein collagen II was downregulated after IL-1β treatment (Figure 2D). In contrast, pretreatment with PT prevented cartilage-decomposing responses, showing that MMP-13 and ADAMTS-5 were reduced and collagen II was elevated (Figure 2D). We further aimed to investigate the signaling pathways in preventing cartilage extracellular matrix degradation by PT. Previous studies have indicated that the NF-κB signaling pathway is involved in the production of inflammatory mediators and MMP degradation.^33,35^ To clarify whether NF-κB activity participates in the anti-inflammatory mechanism of PT under IL-1β exposure, cells were pretreated with PT for 2 h followed by IL-1β treatment for 1 h. The result showed that NF-κB was phosphorylated in response to IL-1β treatment, whereas pretreatment with PT reduced the phosphorylation of NF-κB (Figure 2E). Taken together, the results showed that IL-1β stimulated chondrocytes to produce the cytokine IL-6 through NF-κB activation, leading to degradation of the cartilage extracellular matrix. In contrast, PT reduced the activation of the NF-κB signaling pathway to block the release of inflammatory factors, thereby protecting chondrocytes and maintaining cartilage metabolism in SW1353 cells.

### PT Inhibits LPS/ATP-Stimulated NLRP3 Inflammasome Activation and MMP-13 Production in THP-1 Cells

**Figure 3:**
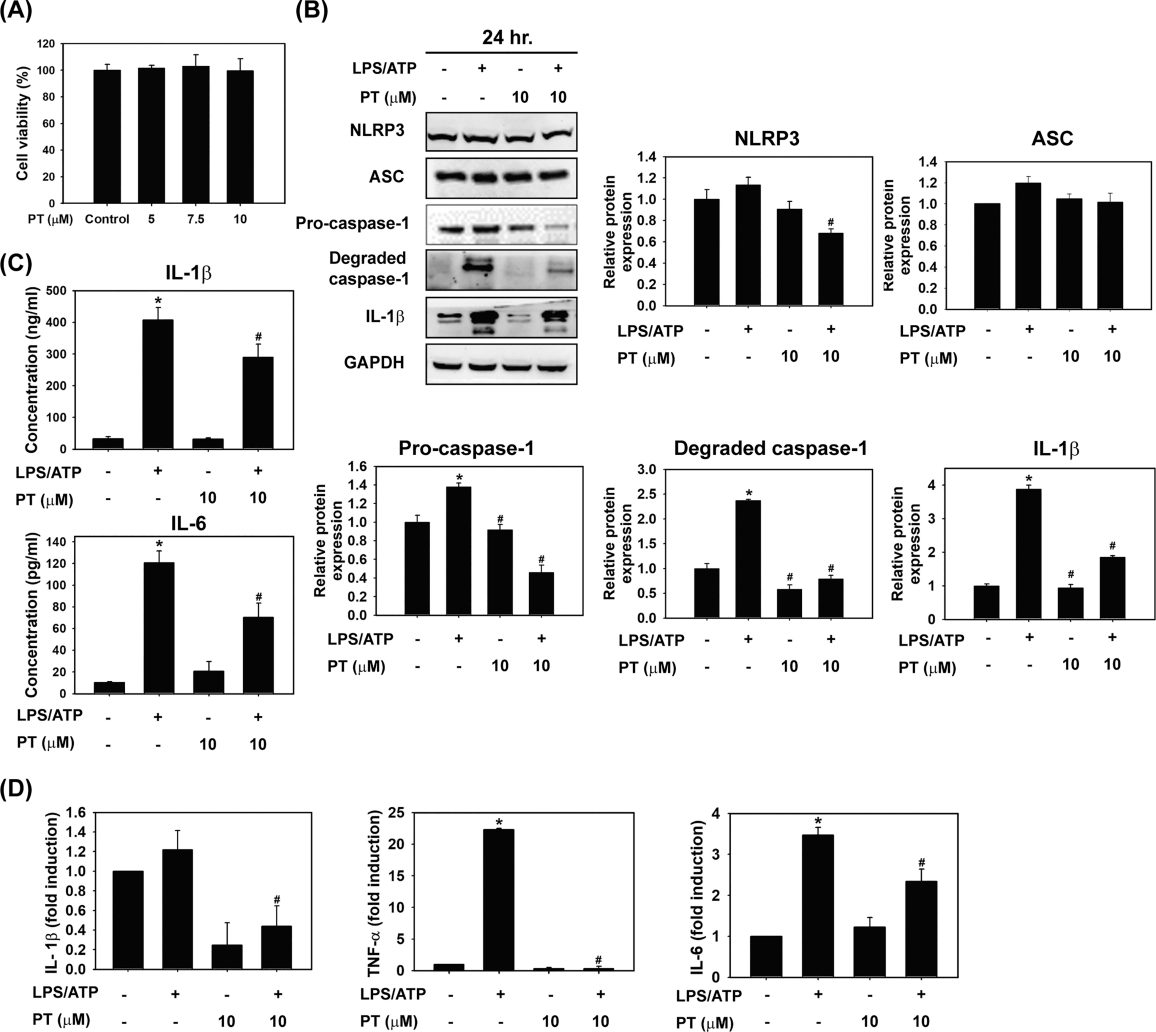
PT suppressed LPS/ATP-induced NLRP3 inflammasome activation and inflammatory cytokines signaling in THP-1 cells. (A) Cell viability was measured by MTT assay in THP-1 cells treated with PT (5, 7.5, and 10 μM) for 24 h. (B) THP-1 cells were treated with PT (10 μM) and/or with LPS/ATP for 24 h. The protein expression of NLRP3, ASC, pro-caspase 1, degraded caspase 1, and IL-1β was detected by Western blot analysis, and their respective quantifications are presented. GAPDH was used as a loading control. (C) The concentration of IL-1β and IL-6 was detected by ELISA. (D) The mRNA expression of IL-1β, IL-6, and TNF-α was analyzed by RT-qPCR after being treated as previously described. All data are presented as mean ± SD from three independent experiments. *p < 0.05 compared with the control groups. ^#^*p* < 0.05 compared with the LPS/ATP groups.

**Figure 4:**
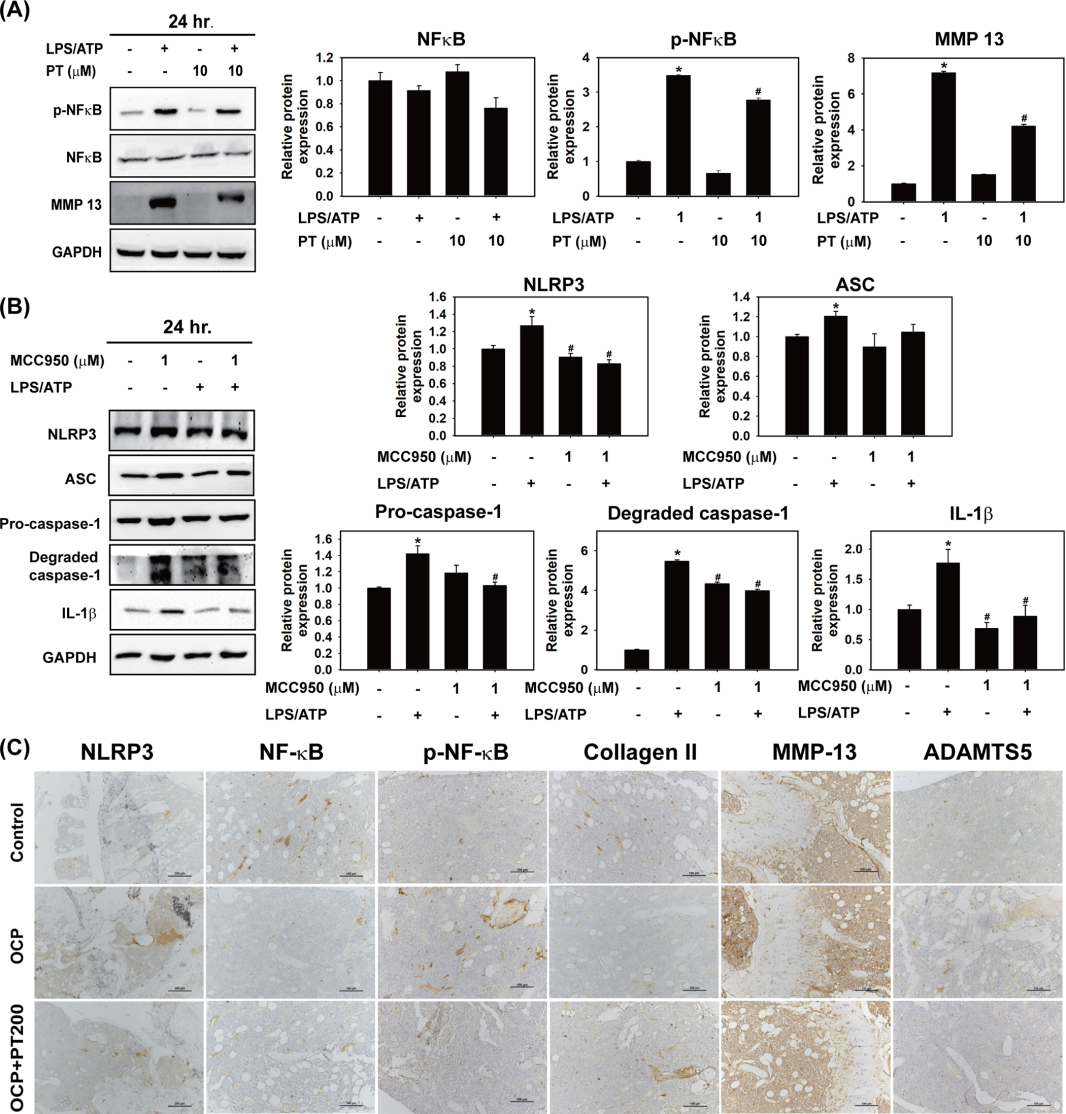
PT inhibited NLRP3 inflammasome and NF-κB activity, which is associated with decreased cartilage degradation in THP-1 cells and the OCP-induced OA model. (A) THP-1 cells were treated with PT (10 μM) and/or with LPS/ATP for 24 h. The protein expression of (A) NF-κB, p- NF-κB, and MMP-13 and (B) NLRP3, ASC, pro-caspase 1, degraded caspase 1, and IL-1β was detected by Western blot analysis, and their respective quantifications are shown. GAPDH was used as a loading control (*n* = 3). All data are presented as mean ± SD from three independent experiments. *p < 0.05 compared with the control groups. ^#^*p* < 0.05 compared with the LPS/ATP group. (C) Immunohistochemistry staining of joint tissues showed the protein expression of NLRP3, NF-κB, p-NF-κB, collagen II, MMP-13, and ADAMTS5 in control, OCP, and OCP + PT 200 groups (original magnification: ×20, scale bar: 100 μm).

Inflammation in the microenvironment is a crucial feature of OA progression. During OA, inflammatory cells, such as macrophages, can release proinflammatory cytokines in the joint via the activated inflammasome pathway.^13^ NLRP3 inflammasomes can be activated by many exogenous and endogenous factors,^36^ including LPS and ATP, both of which were chosen for use in this study. Therefore, we investigated whether PT could inhibit the NLRP3 inflammasome activity and prevent the accumulation of inflammatory cytokines in macrophages. We chose the noncytotoxic concentration of PT for further experiments (Figure 3A). After treatment with LPS/ATP, the expression of NLRP3 increased followed by an increase in the expression of inflammasome-associated proteins ASC, pro-caspase-1, degraded caspase-1, and IL-1β in THP-1 cells (Figure 3B). In contrast, the expression of inflammasome-associated proteins decreased when combined with PT (Figure 3B). In addition, levels of IL-1β and IL-6 significantly increased after LPS/ATP stimulation but decreased under the PT combination (Figure 3C). The mRNA expression analysis showed similar results for the expression of IL-1β, IL-6, and TNF-α (Figure 3D). Furthermore, the level of chondrolytic protein MMP-13 was elevated during LPS/ATP treatment. Nevertheless, PT reduced the expression of MMP-13 in THP-1 cells and the inhibition of the NF-κB pathway (Figure 4A). To confirm the important role of the protective effect of PT through the downregulation of the inflammasome pathway, the NLRP3 inhibitor MCC950 (1 μM) was used to demonstrate the role of inflammation in OA progression. As shown in Figure 4B, the expression of ASC, pro-caspase-1, degraded caspase-1, and IL-1β proteins induced by LPS/ATP decreased when combined with MCC950. These findings indicated that PT, similar to MCC950, acts similarly to inhibit NLRP3 inflammasome activity, thereby eliminating the inflammatory response and MMP-13 accumulation. Furthermore, immunohistochemistry staining revealed a decrease in collagen II and an increase in NLRP3, p-NF-κB, MMP-13 and ADAMTS5 proteins in the OCP groups. However, these changes were ameliorated in the OCP + PT groups (Figure 4C). These results indicated consistent molecular mechanisms in both *in vivo* and *in vitro* experiments, further confirming the PT’s protective mechanism in OA.

### PT Improves Gut Microbiome in OCP-Induced OA

**Figure 5:**
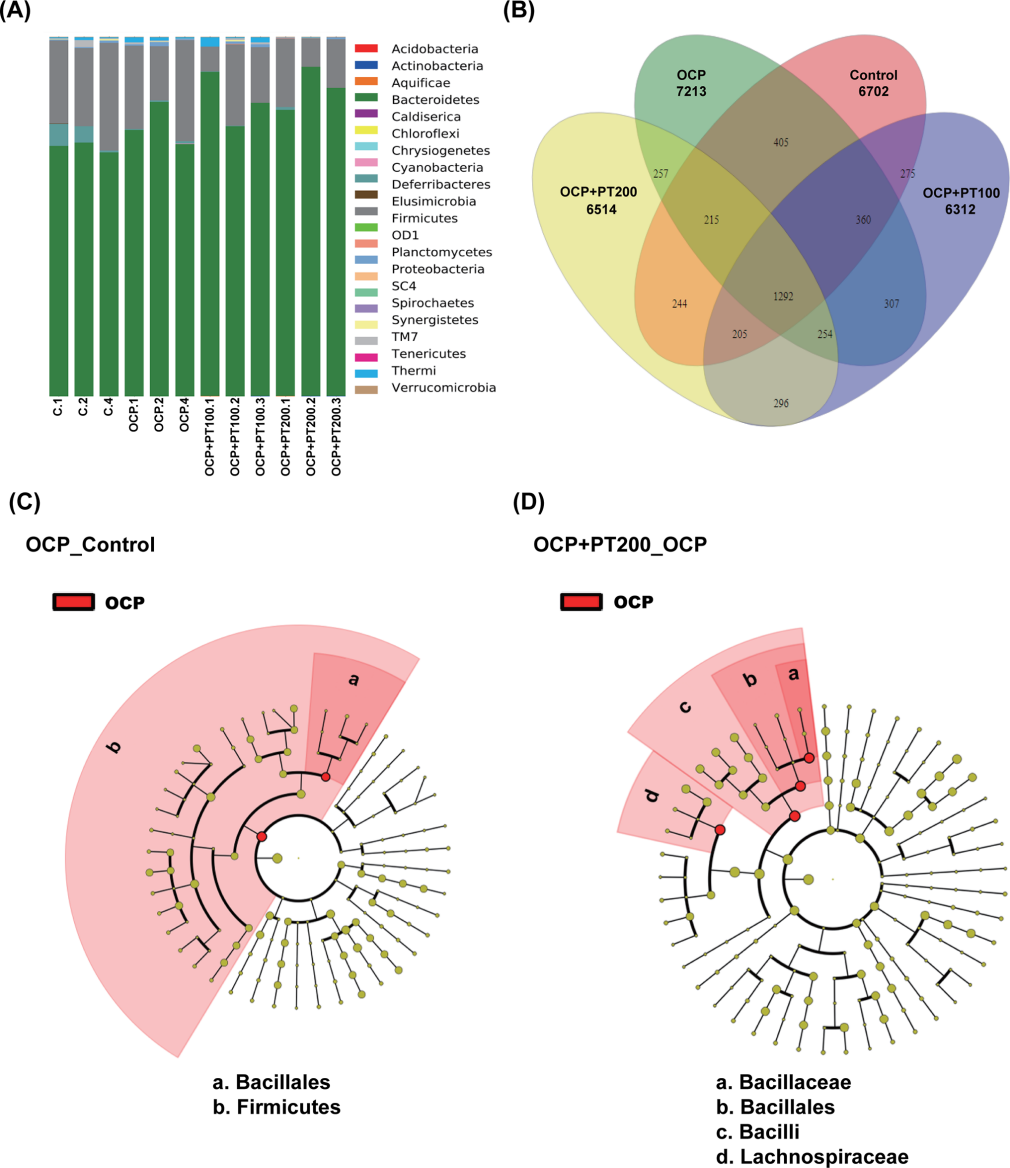
PT changed the proportion of gut microbiota distribution in the OCP-induced OA model. Female C57BL/6 mice were divided into four groups: control, OCP, OCP + PT 100 (mg/kg), and OCP + PT 200 (mg/kg) (*n* = 3 in each group). After 4 weeks of treatment, their feces samples were collected for gut microbiota analysis. (A) The proportion of microbiota in each group was illustrated by taxonomy composition. (B) The correlation of species numbers in the OCP, OCP + PT 100, and the groups with the OCP + PT 200 was expressed by Venn diagrams based on operational taxonomic units (OTUs). (C) LEfSe analysis was used to identify the taxa with the largest differences in abundance in each group. Analysis charts illustrating the difference between the OCP and control groups and (D) the difference between the OCP + PT 200 groups compared with the OCP groups (*n* = 3).

An increasing number of studies suggest that the undesirable changes in gut microbiota may contribute to metabolic syndrome and inflammation, both of which are associated with the induction of OA.^37^ Next-generation sequencing (NGS) was used to evaluate and analyze the impacts of the treatment with OCP and PT on gut microbiota in mice. The taxonomy profiling results displayed the major distribution of the gut microbiota in the control, OCP, OCP + PT 100, and OCP + PT 200 groups (Figure 5A). The ratio of Firmicutes/Bacteroidetes (F/B) in the gut microbiota phyla was decreased in OCP and OCP + PT groups (Table 2). Furthermore, the Venn diagram results, as shown in Figure 5B, indicated that 2272 species were shared between the OCP-treated group and the control group, 1956 species between the OCP + PT-treated group and the control group, and 2018 species between the OCP + PT-treated group and the OCP-treated group. The differences in bacteria between the two groups were further analyzed using linear discriminant analysis (LDA). Figure 5C shows that the OCP-treated groups significantly changed the abundance of Bacillales and Firmicutes compared to the control groups. The changes in Bacillaceae, Bacillales, Bacilli, and Lachnospiraceae were observed in the OCP + PT groups compared to the OCP-treated group (Figure 5D).

**Table 2:** Relative Abundance of Gut Microbiota between Groups at the Phylum Level and F/B Ratio

phylum (%)	control	OCP	OCP + PT 200
Bacteroidetes	69.93 ± 1.77	74.70 ± 4.99	83.72 ± 8.46
Firmicutes	25.30 ± 3.66	22.30 ± 5.40	13.61 ± 5.57
Verrucomicrobia	0.45 ± 0.36	0.78 ± 0.47	1.55 ± 1.24
Deferribacteres	2.95 ± 2.93	1.41 ± 1.45	0.31 ± 0.44
Tenericutes	0.75 ± 0.77	0.34 ± 0.05	0.05 ± 0.01
F/B ratio	**36.18**	**29.85**	**16.26**

**Figure 6:**
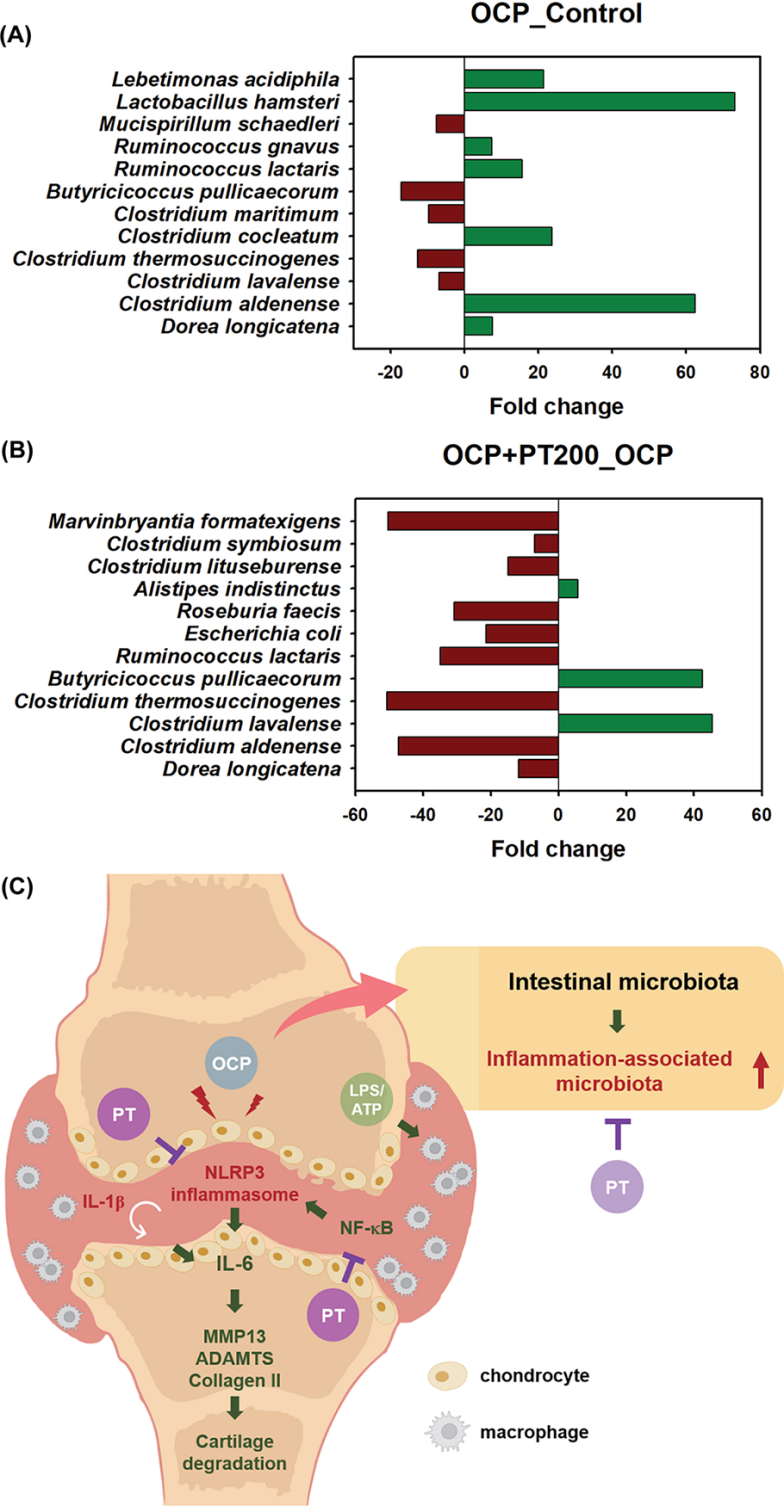
PT improved the composition of gut microbiota in OCP-induced OA. After treatment, mice feces samples were collected for gut microbiota analysis. (A) Metastasis analysis showed the identification of differentially abundant microbiota species in the OCP and the control groups and (B) the OCP + PT 200 and the OCP groups (*n* = 3). (C) A schematic illustration of the prevention effects of PT that reduced OA-induced inflammation and cartilage damage and improved intestinal microbiota. In an OA mouse model, OCP treatment induced cartilage matrix damage, loss of chondrocytes, and narrowed knee joints. In contrast, when combined with PT, it alleviated the damage, improving the overall condition of the knee joints. In SW1353 cells, OCP and IL-1β treatment activated the NLRP3 inflammasome, leading to an increase of inflammatory cytokine IL-6. In addition, LPS/ATP treatment induced the NLRP3 inflammasome activation in THP-1 cells. The activation of NLRP3 inflammasome and NF-κB signaling pathways subsequently decreased collagen II levels while increasing MMP-13 and ADAMTS, resulting in cartilage degeneration. Notably, PT treatment inhibited the activation of the NLRP3 inflammasome in both SW1353 and THP-1 cells and reversed the expression of collagen II and cartilage damage. Furthermore, PT treatment increased the abundance of beneficial bacteria and reduced the abundance of harmful bacteria, improving the overall condition of the gut microbiota in the mice. Taken together, this study has proven that PT protects against OA by inhibiting the activation of NLRP3 inflammasome with health benefits to the gut microbiota.

Figure 6 shows detailed bacterial species analysis with significant differences among groups. In the OCP group compared to the control group, the proportions of *Dorea longicatena*, *Clostridium aldenense*, *Clostridium cocleatum*, *Ruminococcus lactaris*, *Ruminococcus gnavus*, *Lactobacillus hamster*, and *Lebetimonas acidiphila* were significantly increased, whereas *Clostridium maritimum*, *Clostridium lavalense*, *Clostridium thermosuccinogenes*, *Butyricicoccus pullicecorum*, and *Mucispirillum schaedleri* were significantly reduced (Figure 5A). Compared to the OCP group, the OCP + PT 200 groups significantly increased the proportions of *Alistipes indistinctus*, *Butyricicoccus pullicecorum*, and *Clostridium lavalens*, whereas *Dorea longicatena*, *Clostridium aldenense*, *Clostridium thermosuccinogenes*, *Ruminococcus lactaris*, *Escherichia coli*, *Roseburia faecis*, *Clostridium lituseburense*, *Clostridium symbiosum*, and *Marvinbryantia formatexigens* were significantly decreased (Figure 6B). Interestingly, the proportions of *Dorea longicatena*, *Clostridium aldenense*, *Clostridium lavalense*, *Butyricicoccus pullicecorum*, and *Ruminococcus lactaris* presented opposite results in the OCP + PT group compared to the OCP group, suggesting that PT might regulate the composition of microbiota compared to the OA disease model.

## Discussion

In this study, we first determined the protective effects of PT on OA in *in vivo* and *in vitro* models. The highlight of this study is that PT attenuates OA-induced inflammation and reduces cartilage destruction by inhibiting NF-κB and NLRP3 inflammasomes, accompanied by the suppression of the inflammatory cytokine release. In addition, PT also improves the gut microbiota in the OA disease model, which might be associated with preventing OA inflammation and cartilage damage (Figure 6C).

The pathogenesis of OA results from the combined effects of aging, injury, obesity, genetics, mechanical stress, and particularly inflammation.^38^ The use of nontoxic natural products for prevention and disease treatment is the current trend. A previous study demonstrated that curcuminoids were employed as adjunctive treatment for rheumatoid arthritis due to multiple pharmacological effects and fewer side effects than NSAIDs.^15^ On the other hand, a study performed an intra-articular injection of resveratrol, a phenolic compound found in berries, into an OA mouse model. The results show that resveratrol could delay articular cartilage degeneration by balancing HIF-1α and HIF-2α expressions and promoting chondrocyte autophagy, thereby regulating AMPK/mTOR signaling pathway.^39^ Similarly to our study, resveratrol was also reported to induce an inadequate assembly of ASC on the mitochondria and inhibit NF-κB responses, leading to the inactivation of NLRP3 inflammasome.^40−42^ However, the low bioavailability and rapid metabolization made the clinical application of these natural compounds ineffective.^15,39,43^ PT, a dimethyl ether analog of resveratrol, shows better health effects due to its two dimethyl groups, which increase its bioavailability and result in a longer half-life *in vivo*.^21,44^ In particular, several studies suggested that increasing the water solubility of PT significantly improves its oral absorption, thereby promoting the research on the production of highly water-soluble PT-modified compounds and increasing the research potential of PT.^45−47^ Our previous study demonstrated that PT could significantly attenuate renal fibrosis by downregulating NLRP3 inflammasome activation.^12^ Similar to the results in this study, PT affected the regulation of the NLRP3 inflammasome by reducing the expression of TGF-β-induced NLRP3. As a result, ASC and degraded caspase-1 protein expressions were decreased, therefore attenuating the activation of NLRP3 inflammasome.^12^ PT is well established as a safe substance by previous studies.^12,18,24,44,48−50^ In human adults, PT can be safely consumed up to 250 mg per day without damaging any vital organs, such as the liver and kidney.^48,49^ In our previous publication, we used up to 250 mg PT/kg BW to reduce lung tumor multiplicity in mice by 49% without any organ damage or deaths.^51^ Consistent with our study, 200 mg PT/kg BW was safely used as an anticancer agent, as observed in the suppressed tumor growth in a hepatocellular carcinoma mouse model.^52^ In another study, doses of 200 and 500 mg PT/kg BW were orally administered to rats for 28 days without any significant toxicities.^53^ PT is proven to be a potential option in inflammatory OA therapy and prevention in this study. The narrow space of the joint and loss of chondrocytes in OCP-induced OA were recovered into a wider joint space and relatively complete chondrocytes after PT treatment (Figure 1E). More importantly, PT treatment is nontoxic to the liver and kidneys, causing no physical burden, and has beneficial effects on the regulation of microbiota (Figures 1, 5, and 6). Therefore, we strongly suggested that the PT could act as a safe and effective preventive agent against OA.

The inflammation is mediated by immune cells, especially macrophages, in OA pathophysiology.^54^ The infiltration and accumulation of macrophages within the synovium have been proposed as biomarkers of OA progression.^55^ Upon stimulation by DAMPs, macrophages surrounding the cartilage activate inflammasomes such as NLRP3 and release inflammatory factors.^56^ The inflammatory cytokines IL-1β and TNF-α promote the inflammatory environment of synovial joints and perpetuate inflammatory responses by inducing the production of other proinflammatory cytokines.^28^ To prevent the inflammatory response from persistently exacerbating the development of OA, Li et al. suggested that quercetin inhibits IL-1β-induced inflammation and cartilage degradation by suppressing the NLRP3 signaling pathway.^6^ Qian et al. also confirmed that triptolide attenuates the malignant progression of OA by regulating miR-20b/NLRP3.^57^ In our study, PT reduced the inflammatory cytokine IL-6 in chondrocytes and reversed the imbalance of cartilage-decomposing and cartilage-synthesizing proteins in the presence of IL-1β (Figure 2C,D), which was found to be mediated by the inactivated NF-κB pathway (Figure 2E). These results were similar to previous studies showing that PT could alleviate rheumatoid arthritis with a reduction of oxidative stress and the PI3K/Akt/NF-κB signal pathway.^50,58^ Taken together, PT could act as an NLRP3 inflammasome inhibitor, leading to the suppression of NLRP3 inflammasome activation and the release of inflammatory cytokines (Figures 3 and 4).

When the NLRP3 inflammasome was inactivated, the chondrolytic protein MMP-13 was also reduced (Figure 4A), suggesting that the inflammation pathway is associated with the expression of MMP-13. MMPs belong to the ADAMTS family and can regulate the composition of proteoglycans.^8^ MMP-1 is a collagenase, MMP-3 is a potent aggrecanase, and MMP-13 acts against type II collagen.^8^ In particular, MMP-13 is involved in OA cartilage damage by regulating the cartilage proteoglycans aggrecan and fibrillar collagen through inflammatory mediators.^59^ In addition, ADAMT5 is the primary aggrecanase that is activated and able to degrade proteoglycan. When the collagen network begins to break down, irreversible aggrecan and proteoglycan are degraded by activated MMP and ADAMTS.^15^ In our study, inflammatory-cytokine-induced upregulation of MMP-13 and ADAMT5 occurred, whereas the expression of type II collagen in chondrocytes decreased (Figure 2D). However, PT reversed cartilage damage by suppressing inflammation (Figure 4A). These results indicate that PT not only attenuates OA inflammation by inhibiting NF-κB signaling pathway-mediated NLRP3 inflammasome activity but also ameliorates the degradation of collagen.

In addition to the anti-inflammatory effects of PT in OA, PT has also been shown to have beneficial effects on the gut microbiota. The development of OA is accompanied by inflammation, which is closely related to the balance of the gut microbiome.^28^ The correlation between serum levels of bacterial metabolites and joint degeneration serves as a link between gut microbiota dysbiosis and OA.^60^ Alterations of the microbial community by joint-protective nutraceuticals consumption, including PT, could mitigate joint degeneration risk in OA patients.^60^ Chronic local and systemic inflammation are common factors in various diseases and contribute to disturbing the gut microbiome. Imbalances in gastrointestinal bacteria reflect physical condition through persistent and low-grade inflammatory responses.^61^ It is gradually becoming evident that gut microbiome inflammation is induced by proinflammatory cytokines secreting from the immune cells and inflammatory bacterial metabolites.^60^ In addition, the downregulated expressions of Clostridia, Actinobacteria, Enterococci, and Enterobacteria has been found in rheumatoid arthritis patients, indicating a significant relationship between the gut microbiome and joint disease.^27^ Therefore, the correlation between serum levels of bacterial metabolites and joint degeneration serves as a link between gut microbiota dysbiosis and OA.^60^ Additionally, obesity, one of the key risk factors for the development of OA, has been proven to induce inflammation due to changes in the gut microbiome.^62^ Based on these reports, dietary and nutritional supplements have been suggested to ameliorate the gut microbiome to improve the OA.^60^ Polyphenol compounds such as resveratrol and PT are recommended as dietary additions due to their antiobesity effect.^63^ PT can also improve intestinal inflammation and motility disorders, promoting the richness and diversity of probiotics.^64^ Our past research has shown that PT effectively increases the relative abundance of Bacteroidetes and decreases the relative abundance of Firmicutes.^65^ Recently, PT has been reported to reduce inflammation and alter the gut microbiota in rheumatoid arthritis.^27^ Therefore, PT is considered as a great potential candidate to alleviate OA. Indeed, this study showed that the relative abundance of Bacteroidetes was increased and the relative abundance of Firmicutes was decreased in the OCP + PT groups (Table 2, Figure 5A). Additionally, previous reports have claimed that an increased Firmicutes/Bacteroidetes ratio contributes to obesity and intestinal inflammation.^66^ In addition, the results of LEfSe showed that PT lowered the abundance of Firmicutes, such as Bacillaceae, Bacillales, Bacilli, and Lachnospiraceae compared to the OCP group (Figure 5C,D). More importantly, the proportions of *Clostridium aldenense* and *Ruminococcus gnavus*, which have been reported to be related to infection and inflammation,^67,68^ were significantly increased in the OCP group. On the contrary, the proportions of *Clostridium aldenense* and the intestinal inflammation-associated *Marvinbryantia formatexigens*(69) were decreased in PT-combined treatment (Figure 6). Additionally, the proportions of *Mucispirillum schaedleri* and *Clostridium lavalense*, both of which are known for their anti-inflammatory function, were decreased in OCP group,^70,71^ whereas *Clostridium lavalense* and liver protection-associated *Alistipes indistinctus* [50] were elevated in PT-combined groups (Figure 6). Colorectal cancer-associated *Clostridium* species such as *Clostridium cocleatum* and *Clostridium symbiosum* [51, 52] were also increased in the OCP-treated groups but decreased in the PT-combined group (Figure 6). Moreover, *Dorea longicatena* and *Ruminococcus lactaris*, reported to be prevalent in obese patients,^72−74^ were lower in the OCP + PT group compared to the OCP group. The abundance of the emerging probiotics candidate *Butyricicoccus pullicecorum* [56] was also improved after PT treatment (Figure 6). Taken together, the results suggest that OCP may alter the gut microbiota by increasing the abundance of bacteria associated with intestinal inflammation, whereas PT significantly decreases the abundance of these bacteria. These findings demonstrate the efficacy of PT in promoting the healthy development of the gut microbiome and potentially preventing the development of inflammation-related diseases. However, the regulation of PT in most phyla and genera still needs further study.

The underlying mechanism of OA is related to many intricate risk factors, causing the treatment of OA to be a source of numerous dilemmas. Our study is the first to demonstrate that PT could reduce OA inflammation and reverse cartilage damage through NLRP3 inflammasome inhibition. PT can also prevent the vicious cycle of cartilage destruction caused by the persistent inflammatory environment of OA. Additionally, PT provides a therapeutic mechanism and benefits the gut microbiota in the OA mouse model. These results suggest that the PT is an effective candidate for the treatment of OA.

## Abbreviation

ADAMTS: A Disintegrin and Metalloproteinase with Thrombospondin motifs
AGEs: advanced glycation end products
ATP: adenosine triphosphate
BCP: basic calcium phosphate
BUN: blood urea nitrogen
CRE: creatinine
DAMPs: damage-associated molecular patterns
DMEM: Dulbecco’s modified Eagle’s medium
FBS: fetal bovine serum
H&E: hematoxylin and eosin staining
IACUC: Institutional Animal Care and Use Committee
LDA: linear discriminant analysis
LEfSe: LDA effect size
LPS: lipopolysaccharide
MMPs: metalloproteases
MTT: 3-(4,5-dimethylthiazol)-2,5-diphenyltetrazolium bromide
OA: osteoarthritis
OCP: octacalcium phosphate crystals
OUT: operational taxonomic unit
PBS: phosphate-buffered saline (PBS)
PT: pterostilbene
PVDF: polyvinylidene difluoride
SDS-PAGE: sodium dodecyl sulfate-polyacrylamide gel electrophoresis
WHO: World Health Organization
